# Heart Failure with Recovered Ejection Fraction: EGPA-Associated Cardiomyopathy Treated with Benralizumab

**DOI:** 10.3390/jcm15208057

**Published:** 2026-10-16

**Authors:** Laxmi Raj Bangari, Yugant Khand, Vidit Yadav, Aarti Desai, Andrea Muniz, Jose Ruiz, Jose Nativi-Nicolau, Juan Leoni, Parag Patel, Rohan Goswami

**Affiliations:** 1 Department of Heart Failure and Transplant, Mayo Clinic Florida, Jacksonville, FL 32224, USA; laxmirajbangari21@gmail.com (L.R.B.);; 2 Department of Neurology, Mayo Clinic Florida, Jacksonville, FL 32224, USA; 3 Department of Infectious Diseases, Mayo Clinic Florida, Jacksonville, FL 32224, USA

**Keywords:** antibodies, monoclonal, humanized, cardiomyopathies, Churg–Strauss syndrome, eosinophils, heart failure, Interleukin-5 Receptor Alpha Subunit, magnetic resonance imaging

## Abstract

**Background**: We report a case to demonstrate that targeted eosinophil depletion with Benralizumab (anti-IL-5 receptor α therapy) can induce the complete functional and structural reversal of advanced EGPA-associated cardiomyopathy. **Case Presentation**: A 56-year-old woman with a decade-long history of EGPA presented with progressive nonischemic dilated cardiomyopathy and a nadir ejection fraction of 25–30%. Cardiac MRI demonstrated diffuse nonischemic late gadolinium enhancement consistent with eosinophil-mediated myocardial injury. Despite the optimization of guideline-directed medical therapy, complete recovery was not achieved until benralizumab was initiated. Following biologic therapy, the ejection fraction recovered to 50% and severe mitral regurgitation regressed to mild. The exercise capacity was preserved throughout, with a peak VO_2_ of 117% and 111% of predicted before and after biologic therapy, respectively. The patient remains in NYHA Class I at the one-year follow-up. **Discussion**: We describe a case with EGPA-associated non-ischemic cardiomyopathy treated with benralizumab achieving the complete recovery of ventricular function and achieving the complete independence of steroid therapy. **Conclusions**: This case emphasizes the importance of identifying the inflammatory etiology of cardiomyopathy, optimizing GDMT systematically, and considering targeted biologic therapy directed at the underlying disease.

## 1. Introduction

Eosinophilic granulomatosis with polyangiitis (EGPA), formerly Churg–Strauss syndrome, is a rare systemic vasculitis characterized by asthma, eosinophilia, necrotizing vasculitis, and extravascular granulomatous inflammation [1]. Cardiomyopathy and heart failure occur in approximately 15% to 55% of Eosinophilic Granulomatosis with Polyangiitis (EGPA) patients [2,3]. Treatment has historically centered on systemic corticosteroids for remission induction, with cyclophosphamide added in severe or organ-threatening disease and rituximab as an emerging alternative in refractory cases [4]. The introduction of anti-IL-5 biologics, particularly mepolizumab, an anti-IL-5 antibody, and benralizumab, an anti-IL-5 receptor α antibody has since offered a steroid-sparing strategy [4].

### 1.1. EGPA-Associated Cardiomyopathy

The 2025 ESC guidelines define eosinophilic myocarditis histologically and require either endomyocardial biopsy or CMR meeting the updated Lake Louise Criteria, which include a T2-based marker of myocardial oedema. Cardiac involvement in EGPA spans acute eosinophilic myocarditis to chronic inflammatory cardiomyopathy with established fibrosis; the present case represents the latter, with late gadolinium enhancement but no T2-based imaging or histopathology [5].

The two clinicopathological variants of EGPA are antineutrophil cytoplasmic antibody (ANCA)-positive and ANCA-negative phenotype. The ANCA-negative subset is characterized by prominent eosinophilic organ infiltration, including cardiomyopathy with a prevalence of 40–44% [6,7]. EGPA is studied to evolve in three phases: (1) the prodromal phase manifesting as bronchial asthma, allergy, and rhinosinusitis; (2) the eosinophilic phase is characterized by peripheral eosinophilia with the eosinophilic infiltration of specific organs including the lung, heart, and gastrointestinal (GI) tract; and (3) the vasculitic phase—the cardinal manifestation of this phase is peripheral neuropathy occurring in 70.0% of patients, with other features of this phase involving neurological, vascular, and renal involvement [8].

Fifty-percent of deaths in patients suffering from EGPA are related to cardiac diseases [9]. In a meta-analysis of 62 case reports of EGPA with cardiac involvement, cardiac magnetic resonance was done in 46.8% of the patients, and it was abnormal in all. The most common abnormal findings in echocardiography were systolic left ventricular dysfunction (83.9%) and pericardial effusion (37.1%). The most common type of clinical presentation was clinical heart failure (51.6%). Cardiac symptoms, electrocardiographic abnormalities, abnormal biomarkers, and abnormal echocardiography were detected in 82.3%, 68.5%, 77.4%, and 96.8%, respectively [10,11]. The prognosis and severity of EGPA depends on the five-factor score (FFS) given elevated serum creatinine levels, proteinuria, the GI tract involvement, cardiomyopathy, and the CNS involvement [12].

Cardiac involvement in EGPA arises from the direct eosinophil infiltration of the myocardium, toxic granule protein deposition, and coronary vasculitis, a triad that culminates clinically in cardiomyopathy, pericarditis, and progressive heart failure, and that distinguishes this entity from more common causes of nonischemic cardiomyopathy [13]. The cardiac spectrum may include myocarditis, pericarditis, apical aneurysms, conduction abnormalities, and associated cardiomyopathies. The classical pathophysiology of eosinophilic heart disease in EGPA progresses from the myocardial infiltration of cationic proteins causing an acute necrotic phase and intermediate thrombotic phase, and leading to the end-stage fibrotic phase.

According to the screening proposal, 45% asymptomatic patients had cardiac involvement; this suggests that relying on symptoms alone can lead to a delayed diagnosis. Cardiac involvement appears to be associated with more intense disease activity, higher eosinophil counts, and higher CRP and ANCA negativity. CMR is particularly valuable because it can detect subclinical myocardial involvement, including LGE, even when ECG and echocardiography are normal. Therefore, the authors support systematic cardiac screening at EGPA diagnosis and during major flares, with CMR when cardiac involvement is suspected. Most importantly, early detection allows earlier treatment and may improve cardiac outcomes. The main limitations are the retrospective design and non-standardized use of cardiac imaging [14].

### 1.2. Benralizumab—The New Frontier of EGPA Management

The management of EGPA-related cardiac complications involves high-dose glucocorticoids as the universal first-line therapy and cyclophosphamide as the preferred immunosuppressive agent for active cardiac disease. The newer FDA-approved biological agents for EGPA such as Benralizumab or Mepolizumab are currently not recommended over cyclophosphamide or rituximab for remission or severe disease as they were excluded from the two major pivotal trials (MIRRA and MANDARA) [15,16].

IL-5 controls the growth, survival, and activation of eosinophils. It acts through a receptor made of two parts: an α subunit (IL-5Rα) that binds IL-5, and a shared β chain. The available biologics block this pathway in two different ways. Mepolizumab and reslizumab bind IL-5 in the circulation so it cannot reach the receptor. This lowers eosinophil counts but does not remove eosinophils entirely, and some remain in tissue. Benralizumab works differently: it binds IL-5Rα on the eosinophil itself. Its Fc region lacks a fucose sugar, which greatly increases its binding to FcγRIIIa on natural killer cells. The natural killer cells then destroy the eosinophil through antibody-dependent cell-mediated cytotoxicity (ADCC). The result is the near-complete removal of eosinophils from blood, bone marrow, and tissue. This distinction matters in eosinophil-mediated cardiac injury, where the aim is to eliminate the infiltrating cells rather than simply reduce their numbers [17,18].

We report a case to demonstrate that targeted eosinophil depletion with Benralizumab may contribute to the recovery of left ventricular function and the regression of secondary mitral regurgitation in advanced EGPA-associated cardiomyopathy. This study underscores the importance of the direct pathogenic role of eosinophils in myocardial dysfunction and a disease-modifying therapeutic strategy.

## 2. Case Presentation

A 56-year-old woman with a 10-year history of eosinophilic granulomatosis with polyangiitis (formerly Churg–Strauss syndrome) was followed for progressive nonischemic cardiomyopathy. Her disease was ANCA-negative, a phenotype associated with a higher prevalence of cardiac involvement. Her medical history is significant for severe eosinophilic asthma, eosinophilic nephritis, chronic sinusitis with recurrent otitis media, hypertension, type 2 diabetes mellitus (HbA1c 7.2%), Meniere’s disease, mild non-occlusive coronary artery disease, and moderate to severe mitral regurgitation. Two years ago, her lab results indicated elevated eosinophils, with an absolute eosinophil count (AEC) of 620 cells/µL and an eosinophil percentage of 10%.

Coronary angiography in August 2021 revealed only mild non-occlusive distal left anterior descending artery disease, excluding an ischemic etiology. Cardiac magnetic resonance imaging (CMR) performed in the same year showed mildly reduced biventricular systolic function and late gadolinium enhancement (LGE) imaging demonstrated a diffuse, patchy, nonischemic pattern of myocardial fibrosis involving the left ventricle representing sequela of an inflammatory or infiltrative process.

Her at-home medications included Metoprolol 25 mg OD, Sacubitril/Valsartan 24/26 mg PO, and spironolactone 12.5 mg OD, dapagliflozin 10 mg PO. Her symptoms of rhinitis and wheezing were treated with Albuterol nebulization and fluticasone nasal spray, respectively. She was on long-term oral steroids, Prednisone 10 mg. Despite her tolerated guideline-directed medical management, her ejection fraction (EF) fell from 54% in October 2021 to 25–30% by August 2023, and her mitral valve disease progressed from moderate to severe mitral regurgitation. She was referred to our center for further evaluation and Advanced Heart failure management.

### 2.1. Initial Visit and Baseline Cardiac Evaluation

At referral, she was congested and reported orthopnea and exertional dyspnea, corresponding to NYHA class II, ACC/AHA stage C, Stevenson profile B, and INTERMACS profile 6. She was euvolemic by the time of formal evaluation in February 2024. Given the underlying EGPA and prior imaging findings, her cardiomyopathy was attributed to ongoing eosinophil-driven myocardial injury rather than ischemic or purely idiopathic causes. On shared decision making, metoprolol is increased to 25 mg OD, Sacubitril/Valsartan to 49/51 mg, and spironolactone to 12.5 mg along with strict salt and fluid intake optimization. She was started on Prednisone 2 mg for her symptoms of cough and sneezing.

Upon the optimization of GDMT, cardiopulmonary exercise testing (CPET) was performed. The patient completed an 11-minute exercise protocol. The peak oxygen consumption (VO_2_) was 117% of the predicted value, indicating superior functional aerobic capacity. Ventilatory efficiency was within normal limits, demonstrated by a VE/VCO_2_ ratio of 29. The respiratory exchange ratio (RER) and heart rate response were consistent with a maximal, non-limited effort. Clinical course is demonstrated in Table 1.

### 2.2. Therapeutic Intervention

A multidisciplinary decision was made to initiate targeted biologic therapy with Benralizumab (Fasenra) Injection 30 mg subcutaneously once every 4 weeks for first 3 doses, continued by 30 mg once every 8 weeks and Prednisone taper. This therapy was introduced in conjunction with optimized GDMT, including sacubitril-valsartan, metoprolol succinate, spironolactone, and dapagliflozin.

### 2.3. Clinical and Cardiac Recovery

Following the initiation of benralizumab, the patient reported a marked improvement in respiratory symptoms, including the resolution of chronic cough and improved asthma control, allowing corticosteroid tapering. Repeat cardiopulmonary exercise testing in March 2025 showed a peak oxygen consumption of 28.2 mL/kg/min (111% of predicted), with a respiratory exchange ratio of 1.19 confirming maximal effort, an oxygen pulse of 122% of the predicted indicating preserved stroke volume reserve, and a VE/VCO_2_ nadir of 29. Her absolute eosinophil count was 130 cells/µL (2.1%) with current followup of AEC 0cell/uL (0%) (Figure 1).

Echocardiography in March 2025 demonstrated a three-dimensional volumetric ejection fraction of 50% with mild generalized hypokinesis, borderline global longitudinal strain of −16%, left atrial volume index 28 mL/m^2^, and grade 1/3 diastolic dysfunction. Mitral regurgitation had improved from severe to mild. She remained in NYHA class I, ACC/AHA stage C, and Stevenson profile A, without heart failure symptoms, and continued on quadruple GDMT with benralizumab (Figure 2).

## 3. Discussion

This case illustrates several important and clinically meaningful points regarding the diagnosis and management of EGPA associated cardiomyopathy. This case adds to a small but growing body of evidence suggesting that anti-IL-5 pathway therapies are an option in selected patients. In our patient, the timely initiation of targeted eosinophil depletion allowed a sufficient window for myocardial recovery to avoid the progression to advanced heart failure and leading to a transplant, including the need for pretransplant evaluation like cardiac biopsies and its associated procedural risks. Completely eliminating the steroid dependence thereby resulted in the elimination of steroid-related complications that this patient carried over a decade. Lastly, the resolution of severe functional valvular disorders without any valve intervention serves as a reminder that secondary valvular disease in this setting may be fully reversible when the underlying myopathic process is adequately treated.

Eosinophilic cardiomyopathy represents a distinct entity in which myocardial dysfunction is driven by systemic inflammatory activity rather than ischemic or idiopathic mechanisms, necessitating the identification and treatment of the underlying cause. Figure 3 shows that symptomatic cardiac involvement occurs in as many as 27–47% of EGPA cases and represents the leading cause of early mortality and poor long-term prognosis [19]. Notably, cardiac involvement has been reported to be more prevalent among ANCA-negative patients [19], a subgroup into which our patient falls, further contextualizing the severity of her myocardial involvement at presentation.

### 3.1. Multimodal Cardiac Imaging

The early recognition of cardiac involvement is critical, as it represents the primary determinant of mortality in EGPA. This case exemplifies the multimodal cardiac evaluation that should be pursued in any patient with known or suspected EGPA. Echocardiography serves as the frontline tool, identifying wall motion abnormalities, pericardial effusion, valvular involvement, and intracardiac thrombus.

Left heart catheterization is essential in order to exclude obstructive coronary disease, as was performed here, revealing only mild distal LAD disease and confirming a nonischemic etiology [20]. CMR, however, provided the critical additional layer detecting myocardial fibrosis in a nonischemic distribution that echocardiography alone could not characterize, and establishing the inflammatory substrate that ultimately guided therapeutic decision-making. Myocardial edema and pericardial involvement are characteristics of EGPA. Late gadolinium enhancement has been reported in up to 81% of patients with EGPA cardiac involvement, encompassing subendocardial, intramural, transmural, and diffuse patterns [21].

### 3.2. Role of Guideline-Directed Medical Therapy

Achieving maximal GDMT in patients with HFrEF due to systemic inflammatory conditions appears both safe and beneficial. Despite concerns about hemodynamic tolerance in a patient on long-term steroids with borderline blood pressure, our patient ultimately tolerated all four pillars of GDMT, contributing to significant reverse remodeling. Current guidelines recommend pulsed intravenous glucocorticoids for life-threatening cardiac manifestations of EGPA, and cyclophosphamide for remission induction in patients with severe cardiac, gastrointestinal, or neurological involvement [22].

Our patient had been maintained on long-term oral corticosteroids without cyclophosphamide, a management strategy that suppressed but did not eliminate eosinophilic end-organ activity. This raises a clinically important question: whether earlier escalation to targeted biologic therapy, rather than prolonged corticosteroid dependence, might have attenuated the degree of myocardial injury she sustained over the preceding years. While this question cannot be answered retrospectively, it has direct implications for how EGPA patients with cardiac involvement should be monitored and when the threshold for biologic initiation should be lowered.

### 3.3. Role of Benralizumab and Steroid Dependence

The temporal association between the initiation of benralizumab and rapid cardiac recovery suggests a potential disease-modifying role beyond conventional immunosuppression. The depletion of eosinophils through the ADCC mechanism described above removes the effector cell driving myocardial injury, which corticosteroids suppress but do not eliminate (Figure 4).

Prior to biologic therapy, GDMT optimization had improved LVEF from 25–30% to 35–40% over approximately 6 months, but incomplete recovery. Following benralizumab initiation, LVEF rose to 50% within weeks to a couple of months, the resolution of mitral regurgitation to mild [4]. While causality cannot be established from a single case, this trajectory is temporally compelling and mechanistically plausible.

A small number of case reports have previously described cardiac improvement following benralizumab in eosinophil-mediated cardiac disease. One described a patient with eosinophilic cardiogenic shock who showed the complete clearance of myocardial eosinophil infiltration on biopsy 26 days after treatment with steroids and benralizumab [23]. Another case report of a patient with refractory hypereosinophilic syndrome reported a partial/transient response with mepolizumab, which was then treated with benralizumab, achieving a complete response [24].

Evidence for benralizumab in EGPA with cardiac involvement remains confined to case reports and small series. A recent real-world series of three patients with severe, steroid-refractory ANCA-negative EGPA reported rapid and sustained eosinophil depletion in all cases, the complete resolution of cardiac lesions in one patient, and glucocorticoid discontinuation in two [25]. In an earlier review of 41 patients with EGPA treated with benralizumab, oral corticosteroids were reduced to 10 mg/day or less in every case and discontinued in more than 40%, with reported efficacy in patients whose cardiac and neuropathic complications had proved refractory to mepolizumab [26]. Our patient extends these observations by documenting the recovery of ventricular function and regression of secondary valvular disease over a four-year period, with the endpoints not captured in the existing reports.

Long-term oral steroids carry a heavy cost, including diabetes, hypertension, osteoporosis, and infection. Coming off them completely, rather than simply taking less, is therefore a meaningful treatment goal. In the phase 3 MANDARA trial, benralizumab and mepolizumab both allowed most patients to cut their steroid dose by at least half. However, the complete withdrawal of oral steroids was achieved in 41% of patients on benralizumab compared with 26% on mepolizumab [16]. In a subsequent analysis of the same trial, 33 of 70 benralizumab-treated patients versus 20 of 70 mepolizumab-treated patients completely withdrew from oral glucocorticoids (hazard ratio 1.84, 95% CI 1.06–3.27), and 17 of 70 versus 7 of 70 sustained complete withdrawal (hazard ratio 2.97, 95% CI 1.26–7.77) [27]. The significant reduction in steroid burden with benralizumab may have also had independent cardiac and metabolic benefits, including the attenuation of steroid-related cardiomyopathy, hypertension worsening, and steroid-induced diabetes mellitus.

These benefits appear durable. In the two-year open-label extension of MANDARA, complete steroid withdrawal was maintained and continued to improve, reaching 43.9% of patients who received benralizumab throughout, with low relapse rates in both groups [28]. Patients switched from mepolizumab to benralizumab showed a further fall in blood eosinophil count from 70 to 20 cells/µL within four weeks, along with improved steroid sparing [28]. This observation supports the mechanistic distinction described earlier: residual eosinophils persisting on ligand-directed therapy were cleared only after receptor-directed depletion. Cardiac endpoints were not assessed in the extension study.

Ultra-long-acting IL-5 blockade is a further option that should be taken into account. Depemokimab is a humanized anti-IL-5 monoclonal antibody that enhances IL-5 binding and has an extended half-life, which allows subcutaneous dosing every 26 weeks, compared with every 4 weeks for mepolizumab. Like mepolizumab, depemokimab neutralizes circulating IL-5 rather than depleting eosinophils through ADCC. Its efficacy and safety in relapsing or refractory EGPA are being compared with those of mepolizumab in the ongoing phase 3, randomized, double-blind, non-inferiority OCEAN trial (NCT05263934) [29,30].

Our case builds on these prior reports in several meaningful ways. The diagnosis was specifically eosinophilic granulomatosis with polyangiitis (EGPA), rather than hypereosinophilic syndrome. Cardiac recovery was longitudinally documented over four years using serial echocardiography and cardiopulmonary exercise testing. Importantly, the patient achieved complete steroid independence. The sequential application of guideline-directed medical therapy followed by benralizumab also provides a clearer understanding of the optimal therapeutic window and the incremental contribution of each intervention to sustained cardiac recovery.

A growing body of evidence supports IL-5– and IL-5Rα–directed therapy in eosinophilic myocarditis, much of it in the context of EGPA. The reported series describe a favorable safety and clinical response, though the literature remains limited to case reports and small single-center cohorts with heterogeneous diagnostic criteria and short follow-up [31,32].

The growing body of evidence supporting biologic therapy in EGPA has focused predominantly on respiratory outcomes, with cardiac endpoints remaining a secondary consideration at best. Screening algorithms for cardiac involvement in EGPA have been proposed, including baseline CMR at diagnosis with repeat imaging and periodic biomarker assessment [19]. What remains absent is evidence on how the heart responds to treatment. The pivotal biologic trials did not incorporate protocolized cardiac endpoints, and prospective data on LVEF trajectories following IL-5 or IL-5Rα–targeted therapy are lacking. No threshold has been established for initiating biologic therapy on cardiac rather than purely respiratory indications. Prospective studies incorporating cardiac endpoints are needed.

### 3.4. Reversibility of Valve Disorders with Disease-Modifying Therapy

Current guidelines recommend the consideration of mitral valve intervention in patients with severe functional MR and LVEF below 60% who remain symptomatic despite optimal medical therapy [33]. In our patient, the complete resolution of MR from severe Stage C2 to mild without any procedural intervention reinforces the principle that functional MR in the context of dilated cardiomyopathy should be managed with aggressive treatment of the underlying myopathic process before valve surgery is considered. Had intervention been pursued at the nadir of her disease, it would have been unnecessary in retrospect and potentially harmful.

### 3.5. Safety Profile of Benralizumab in EGPA

Our patient tolerated benralizumab without any adverse effects throughout the observation period, consistent with the generally favorable safety profile reported in the literature. Across available studies, benralizumab demonstrated safe and tolerable treatment in patients with EGPA. In a retrospective study by Nanzer et al., involving 70 patients treated for two years, no serious adverse events were noted [34]. Similarly, in a monocentric study by Desaintjean et al., patients treated with mepolizumab or benralizumab along with glucocorticoid revealed no significant adverse events or anaphylaxis, with headache as the most reported side effect [35].

In contrast, real-world data from Bettiol et al. demonstrated patients experienced adverse reactions leading to treatment discontinuation and reported pneumonia, fractures, and asthma exacerbations as most common adverse events but non serious manageable side effects [36]. The discrepancy likely reflects differences in patient selection, follow-up duration, and reporting methodology between controlled studies and real-world cohorts.

While emerging evidence supports the efficacy of benralizumab therapy in systemic and tissue infiltrated eosinophilic disorders, their impact on cardiac involvement remains understudied. The existing studies are mainly case reports or Case series, the absence of prospective data limits the ability to establish causality, in defining treatment timing and identifying the predictors and prognostic indictors for cardiac recovery.

## 4. Conclusions

We describe a patient with longstanding EGPA-associated nonischemic cardiomyopathy who an experienced 50% recovery of left ventricular ejection fraction, with the regression of severe functional mitral regurgitation to mild, and who achieved complete independence from corticosteroids following optimization of guideline-directed medical therapy and initiation of benralizumab. Exercise capacity, already normal before biologic therapy (peak VO_2_ 117% of predicted), remained preserved thereafter (peak VO_2_ 111% of predicted). This case underscores the importance of identifying the inflammatory etiology of cardiomyopathy, optimizing GDMT systematically, and considering targeted biologic therapy directed at the underlying disease mechanism as a potentially transformative intervention. Further prospective studies are warranted to evaluate the cardiac outcomes of anti-IL-5Rα therapy in EGPA.

## 5. Limitations

This case documents complete steroid withdrawal after a decade of dependence, sustained eosinophil depletion, and the recovery of ventricular function with regression of mitral regurgitation over four years. Its limitations are real. No troponin, repeat CMR, or biopsy was obtained during the decline, so the attribution to active eosinophil-mediated injury is inferential. GDMT was optimized over the same period, and we cannot say how much of the recovery belongs to eosinophil depletion. Earlier echocardiograms were performed at outside institutions and reported as ranges, which limits the precision when compared with the three-dimensional measurement obtained at our center. As a single case, this can only generate hypotheses; a retrospective cohort from a larger EGPA population would better define patient demographics, cardiac risk factors, treatment timing, and predictors of myocardial recovery.

## Figures and Tables

**Figure 1:**
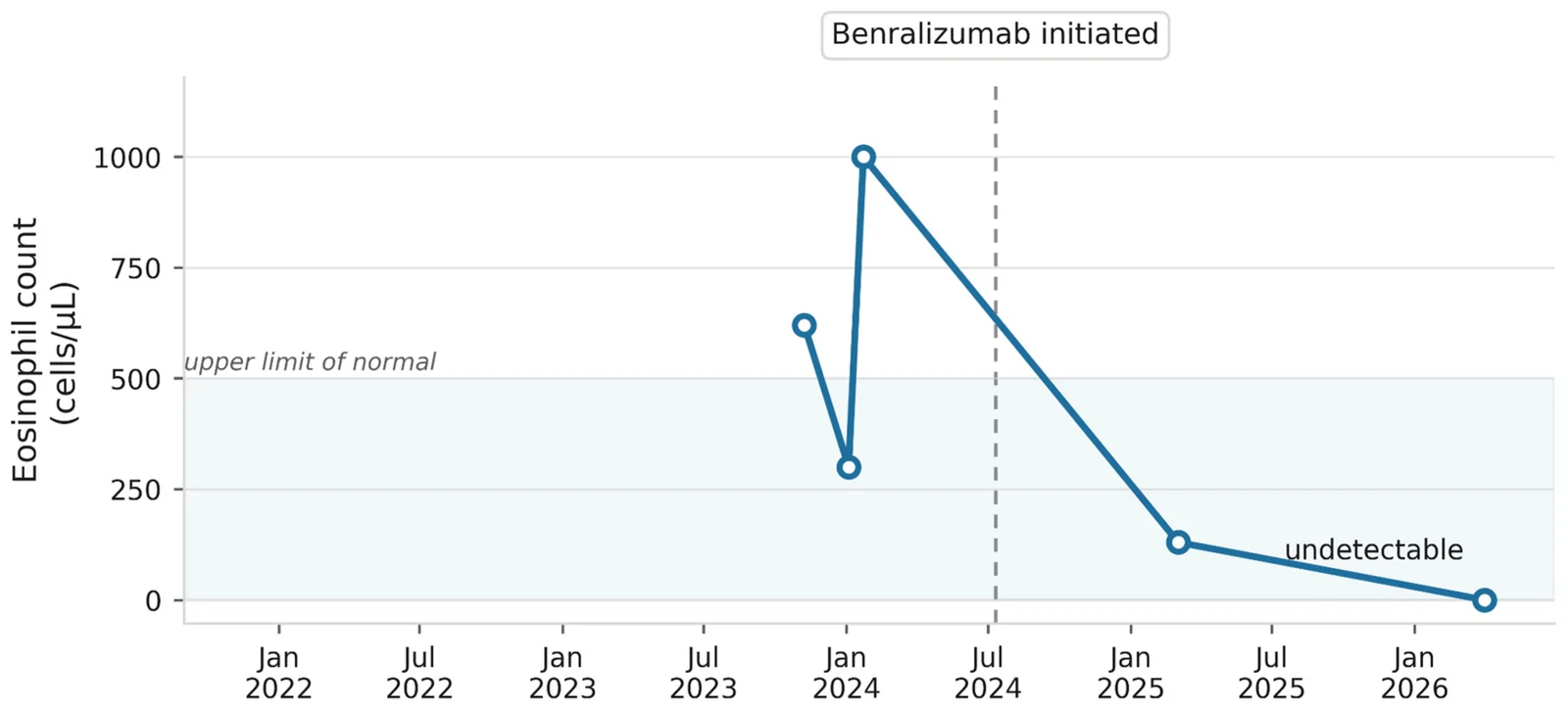
Eosinophil count over time.

**Figure 2:**
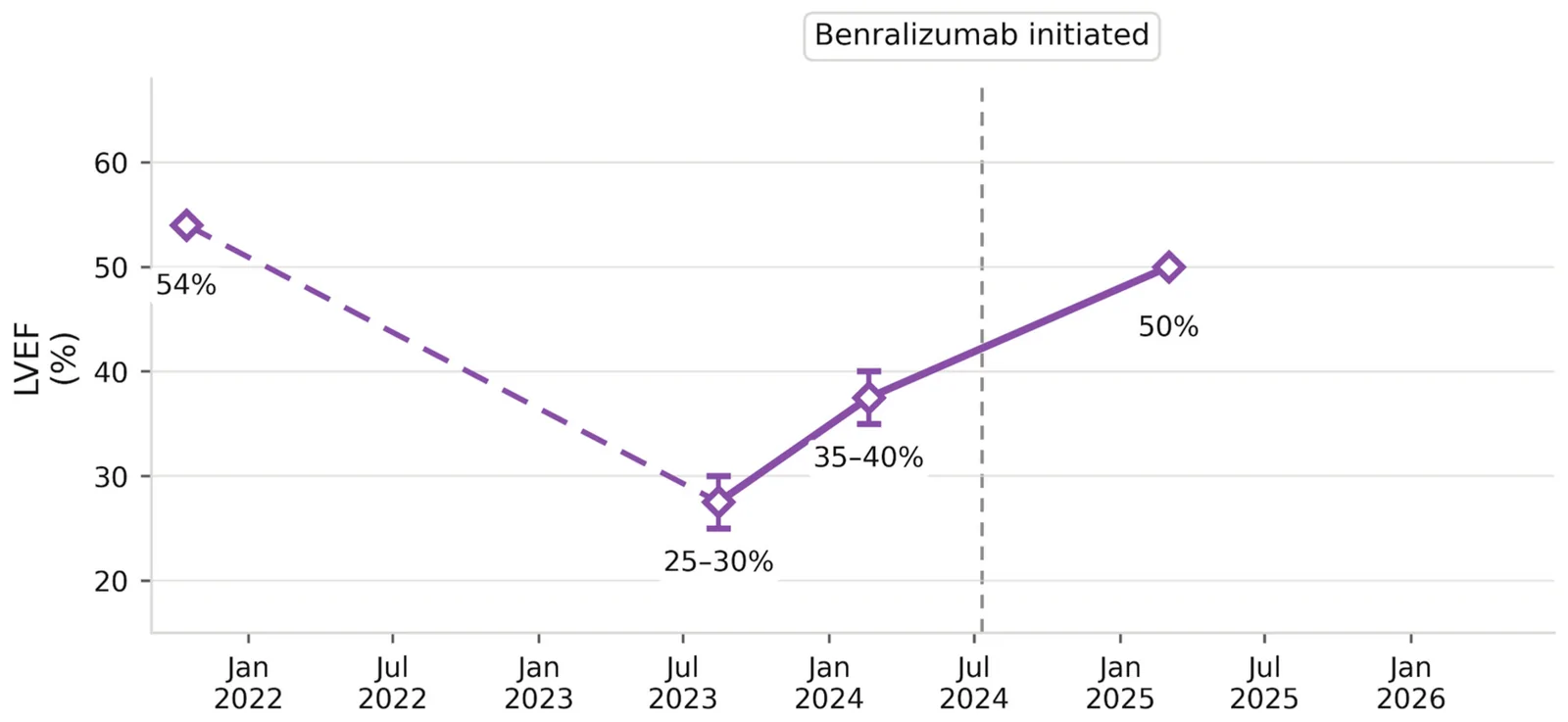
LVEF over time.

**Figure 3:**
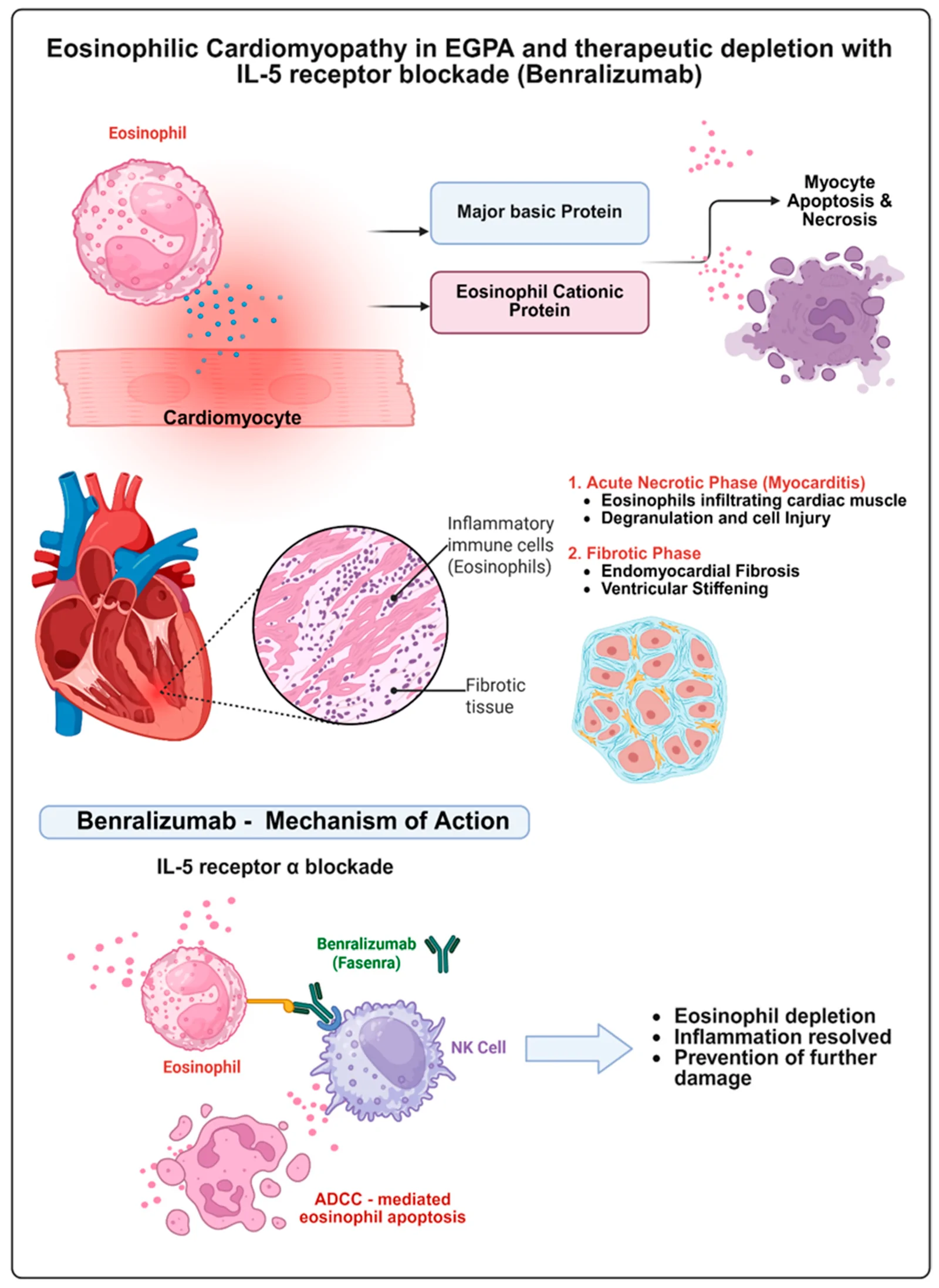
Eosinophilic cardiomyopathy in EGPA and therapeutic depletion with IL-5 receptor blockade (Benralizumab).

**Figure 4:**
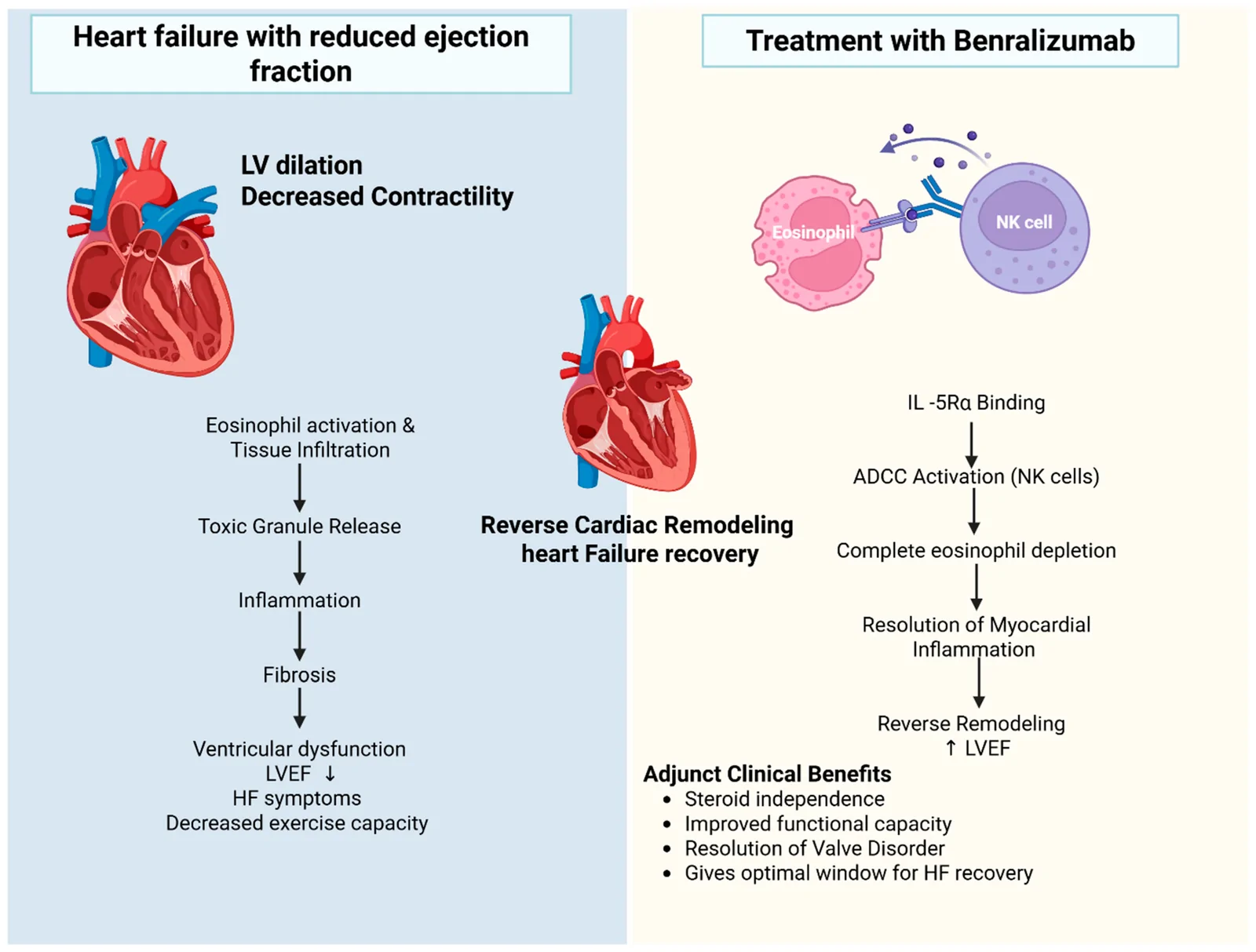
Cardiac recovery in HFrEF with benralizumab. Proposed mechanism of cardiac recovery in HFrEF following IL-5Rα blockade with benralizumab. The pathways illustrated are hypothesized and are not directly demonstrated in this case.

**Table 1:** Clinical course and therapeutic response in EGPA cardiomyopathy.

Clinical Course	Time Point	NT ProBNP	Key Events	Medications
*Baseline Evaluation*	October 2021	-	LVEF: 54% CMR: Late gadolinium enhancement Moderate mitral regurgitation (MR)	Prednisone 20 mg
*Clinical deterioration*	August 2023	451 pg/mL	LVEF: 25–30% Progression to severe MR Absolute eosinophil count (AEC): 620 cells/µL Eosinophils: 10%	GDMT Initiation: Metoprolol 25 mg Spironolactone 12.5 mg Sacubitril/valsartan 24/26 mg Dapagliflozin 10 mg
*Advanced heart failure evaluation*	February 2024	182 pg/mL	CPET—Peak VO_2_ 117%—Preserved Functional capacity LVEF: 35–40% AEC: 1000 cells/µL	Up-titration Sacubitril/valsartan 49/51 mg
*Biologic therapy*	July 2024	-	Persistent eosinophilic activity despite standard therapy	Benralizumab 30 mg Initiated Prednisone taper 2 mg Spironolactone 25 mg
*Cardiac recovery*	March 2025	199 pg/mL	LVEF improved to 50% Mitral regurgitation reduced to mild CPET—Peak VO_2_ 111% of predicted AEC: 130	Maintenance therapy with Benralizumab + GDMT Complete cessation of Prednisone
*Latest follow-up*	April 2026	195.1 pg/mL	0 cells/µL (0.0%)	Benralizumab maintenance; no Prednisone therapy

## Data Availability

De-identified data supporting the findings of this study are available from the corresponding author upon reasonable request.

## Abbreviations

The following abbreviations are used in this manuscript:

EGPA	Eosinophilic Granulomatosis with Polyangiitis
IL-5	Interleukin-5
IL-5Rα	Interleukin-5 Receptor Alpha
AEC	Absolute Eosinophil Count
CMR	Cardiac Magnetic Resonance Imaging
LGE	Late Gadolinium Enhancement
GDMT	Guideline-Directed Medical Therapy
HFrEF	Heart Failure with Reduced Ejection Fraction
CPET	Cardiopulmonary Exercise Testing
VO_2_	Peak Oxygen Consumption
NYHA	New York Heart Association
AHA	American Heart Association
INTERMACS	Interagency Registry for Mechanically Assisted Circulatory Support
METs	Metabolic Equivalents
MR	Mitral Regurgitation
